# Variation in referrals from primary care to scheduled paediatric services in North and East Scotland -a cross-sectional study

**DOI:** 10.1186/s12913-021-06986-0

**Published:** 2021-09-20

**Authors:** Smita Dick, Ryen Crabb, Claire McFaul, Clare MacRae, Philip Wilson, Steve Turner

**Affiliations:** 1 Child Health, Royal Aberdeen Children’s Hospital, University of Aberdeen, AB25 2ZG Aberdeen, UK; 2 Usher Institute, University of Edinburgh, Edinburgh, UK; 3 Centre for Rural Health, University of Aberdeen, Inverness, UK

**Keywords:** Primary Health Care, General Practice, Referral and Consultation, Pediatrics, Health Services

## Abstract

**Background:**

Factors contributing to decisions to refer children for scheduled appointments at medical paediatric outpatient clinics are not well understood. Our aim was to describe practice-level characteristics associated with referrals to general paediatric clinics.

**Methods:**

In this cross-sectional study the setting was general practices in three health boards in Scotland, NHS Grampian, NHS Highland and NHS Tayside The outcome was average annual number of referrals per 1000 children between 2011 and 2017. Univariate and multivariate analyses related the outcome to practice characteristics. For each practice the following characteristics were determined: distance from hospital; area deprivation; number of children registered; presence of ≥ 1 general practitioner with a child health interest and practice ownership.

**Results:**

There were 62 practices in NHS Grampian, 63 in NHS Highland, and 65 in NHS Tayside; representative annual number of referrals to paediatric clinics per capita were 22, 34, and 35/1000 respectively. In the multivariate model, the number of referrals was inversely related to number of children in the practice (0.8 % fall per 1000 children [95 % confidence interval, CI, 0.5, 1.1]) and was higher from practices in the more deprived areas by a mean 55 % [95 % CI 9, 121] compared to less deprived areas. The number of referrals from a practice rose by 0.91 % [95 % CI 0.86, 0.97] for each additional partner in the practice.

**Conclusion:**

Some practice-level characteristics were related to the standardised number of referrals, and associations differed between regions.

## Introduction

There is a well-recognised call to transform child health services in the UK and adopt a more integrated model of care [1, 2]. The focus on transformation has been greater for unscheduled care than for scheduled care, and this reflects the pressure on secondary care services from the rising numbers of short stay unscheduled admissions to paediatric wards across the UK [3, 4]. Primary care decision-making in the context of an unscheduled paediatric referral is increasingly well understood [5] but factors contributing to decision-making prior to a scheduled referral to paediatric services are less well understood.

Health care in the UK is provided free at the point of delivery. Registration with a single general practice by patients is required. General Practitioners (GPs) provide both scheduled and unscheduled primary care health care, and act as a gate-keeper to secondary care services. Scheduled referrals to secondary care services almost always come from scheduled GP care, with small numbers of referrals originating from other referral sources such as unscheduled GP services, clinicians working in Emergency Departments and other hospital-based healthcare professionals.

There is a limited literature describing factors associated with scheduled referrals to hospital. For all patient age groups, characteristics of the clinician, patient and practice may be important to decision-making prior to referral, but these traits may explain a minority of variation in referral rates and intrinsic psychological variable of the GP’s may be important [6]. The likelihood of referral may be related to the gender and training of a clinician [7, 8] and also whether the patient is involved in shared-decision making [9]. In a service improvement project from Wales, characteristics of the area or the practice did not appear to influence the rates and a fall in referral rates lasted for the intervention period only [10]. Specific to referral to paediatric services, factors important in decision-making prior to scheduled referral may include the referrer’s knowledge of the underlying condition [11] and parental request [11, 12].

The relationship between the number of patients registered with the practice and referral rates is complex, and higher numbers of patients have been associated with both higher and lower referral rates [6]. The possible explanation could be that an increase in patient numbers might be associated with greater number of GPs within the practice, increasing the likelihood of available paediatric expertise and thus potentially reducing referral rates.

Scheduled hospital outpatient visits are rising [13] and reasons for this are not understood, but may include parent/carer health seeking behaviour, primary care staff unfamiliarity with some child health conditions, pathways which encourage referral and increasing complexity of patients. We anticipated that practice characteristics associated with referral numbers would vary by region, so we obtained data for three adjacent regions (Scottish health boards) to gain insight into any relevant characteristics. Knowing these characteristics could inform integrative strategies where both primary and secondary care teams provide shared care in the community [1, 2]. Our hypothesis was that characteristics of a primary care practice would be related to the number of referrals of children to medical outpatient departments.

## Methods

### Aim

To investigate which practice characteristics might be related to the number of scheduled referrals to paediatric clinics.

### Study design

In this cross-sectional study we obtained the number of scheduled referrals from each primary care practice to medical paediatric clinics between 2011 and 2017 from three adjacent health boards (NHS Grampian, NHS Highland and NHS Tayside). Data collected over several years were used in order to smooth out years with exceptionally high or low referral numbers (likely to occur in practices with smaller numbers of registered patients). The outcome was average number of referrals 2011–2017 per practice per 1000 patients aged under 15 years registered with that practice. This outcome was related to characteristics of individual practices already identified as being linked to number of referrals [6] or which we reasoned may influence decision-making leading to a referral to paediatric outpatients.

### Setting

Characteristics of the three health boards which contributed data are shown in Table 1. There is only one inpatient unit in NHS Tayside (located in Dundee) and in NHS Highland (located in Inverness). Grampian has two inpatient units, Royal Aberdeen Children’s Hospital (RACH, for Aberdeen City and Aberdeenshire) and Dr Gray’s Hospital in Elgin (DGH, for Moray). For the purpose of this analysis, only NHS Grampian data for referrals to RACH were considered and practices referring to DGH were excluded. The units in Aberdeen and Dundee offer some tertiary services; referral numbers in this study included only referrals to medical paediatrics and not subspecialty paediatrics. None of the health boards had a comprehensive pathway for scheduled referrals, although for some common conditions some boards had web-based documents which indicate when a scheduled referral may be considered.

**Table 1:** Details of the three Health Boards which contributed data. The population data were obtained from Mid population statistics 2013^a^

NHS health board	Number of children in 2013 aged < 15 years, (% all children in Scotland)	Area covered by NHS Health board in square miles (children/square mile)	Median deprivation quintile (IQR, 1 = most deprived))	Number of practices	Mean (SD) and *median [IQR, minimum-maximum]* number of referrals/1000 children/year (averaged over 2011–2017).
NHS Grampian	91,711 (11 %)	3360 (27)	3 (2,4)	62	22 (7) *22 [18–24, 4–52]*
NHS Highland	50,604 (6 %)	12,507 (4)	3 (2,4)	63	119 (232) *34 [12–132, 2-1562]*
NHS Tayside	63,335 (7 %)	2986 (21)	3 (2,4)	65	35 (8) *34 [30–40, 22–74]*

^a^https://www.nrscotland.gov.uk/statistics-and-data/statistics/statistics-by-theme/population/population-estimates/mid-year-population-estimates/mid-2013

### Practice characteristics

The following characteristics were determined for each practice:

1. GP with a child health interest. Each practice web site was examined in Dec 2018 to identify the number of GPs with a child health interest. This was defined as stating an interest in child health and/or having either Diploma of Child Health, Membership of the Royal College of Paediatrics and Child Health.
2. The distance from each practice to the hospitals in Aberdeen, Inverness and Dundee was calculated by entering postcodes into AA Route planner (https://www.theaa.com/route-planner/route). Where a practice had two or more sites, the site furthest from the hospital was used. NHS Tayside and Highland (but not Grampian) offer clinics in venues other than the hospital, but for the purposes of this analysis the distance to hospital was used. Distance was categorised into tertiles for analysis to allow for the considerable distances between some practices and hospital.
3. Deprivation of the practice community. Quintile of deprivation was derived from the 2016 Scottish Index of Multiple Deprivation (SIMD) using the practice postcode. The two least affluent SIMD quintiles and the two most affluent SIMD quintiles were merged to create three categorises containing an approximately similar number of practices for analysis.
4. “2 C status” (yes or no). In Scotland most practices are owned by the GP partners, but some are owned by the health board (called “2 C” practices), often due to difficulties in recruiting staff. https://www.isdscotland.org/Health-Topics/General-Practice/Quality-And-Outcomes-Framework/Glossary.asp.
5. The number of children aged < 15 years in each practice population was obtained from https://www.isdscotland.org/Health-Topics/General-Practice/Workforce-and-Practice-Populations/.
6. The number of doctors in the practice was obtained from the practice web site in Dec 2018. This included partners, salaried partners and part time partners but not locums.

### Statistical analysis

For each health board, univariate analyses using student’s T, Mann-Whitney, Kruskal-Wallis or Spearman’s tests were used to relate the outcome (i.e. number of referrals/1000/year) to explanatory variables of interest (i.e. the six practice characteristics described above). Where the p value from the univariate analysis was < 0.2 the explanatory variable was included in a linear multivariable model for each board. Finally, data from all three boards were combined. Correlations between explanatory variables were sought and the outcome was related to all explanatory variables. IBM/SPSS version 25.0.0 was used and *p* < 0.05 in the multivariate analyses was considered significant.

## Results

### Practice details

There were 62 practices in NHS Grampian, 63 in NHS Highland and 65 in NHS Tayside, Table 1. The median number of referrals per practice was similar for NHS Highland and NHS Tayside and relatively lower for NHS Grampian (the latter reflecting the availability of more subspecialty clinics in NHS Grampian). The outcome was not available or was zero in three practices from Grampian and Highland and two in Tayside. Information on GPs with a child health interest was not available from 12 practices in Grampian, 21 in Highland and 11 in NHS Tayside. Table 2 presents characteristics of the practices where the outcome was available.

**Table 2:** Results from univariate analyses for factors potentially associated with the prevalence of referrals per 1000 0-14 year olds per annum^a^

		Grampian (***n*** = 62)				Highland (***n*** = 63)				Tayside (***n*** = 65)			
		N	mean (SD) referral rate/1000/y	Median, [IQR] (range) referral rate/1000/y	***p*** value	N	mean (SD) referral rate/1000/y	Median, [IQR] (range) referral rate/1000/y	***p*** value	N	mean (SD) referral rate/1000/y	Median, [IQR] (range) referral rate/1000/y	***p*** value
**GP with child health interest**	Yes	21	25 (9)	23 [21-27] (14, 52)	0.015	13	231 (429)	57 [9, 299] (2, 1562)	0.809	28	37 (10)	24 [30, 41] (23, 74)	0.198
	No	29	20 (6)	19 [17-24] (4, 31)		29	101 (138)	49 [13, 147] (2, 566)		26	34 (5)	35 [30, 37] (22, 45)	
	Missing	12				21				11			
**Distance from hospital in tertiles (1 = closest)**	1	19	25 (8)	23 [20-28] (15, 52)	0.153	21	76 (79)	34 [12, 147]	0.993	21	37 (10)	35 [31, 40] (25, 74)	0.423
	2	20	22 (6)	22 [17, 26] (17, 28)		21	121 (185)	38 [10, 113]		22	34 (6)	31 [30, 41] (25, 47)	
	3 (ref)	20	20 (7)	20[17, 26] (4, 31)		21	160 (350)	34 [13, 126]		20	34 (8)	33 [29, 40] (22, 56)	
	Missing	3				0				2			
**Deprivation Index (1 = most)**	1 and 2	7	28 (5)	27 [25-31] (22, 37)	0.013	16	112 (104)	25 [86, 171]	0.171	24	34 (5)	34 [30, 38] (25, 45)	0.474
	3	10	25 (11)	23 [19, 28] (14, 52)		31	132 (304)	29 [10, 99]		17	37 (13)	34 [30, 43] (22, 73)	
	4 and 5	39	20 (7)	23 [19, 28] (14, 52)		16	100 (161)	32 [6, 137]		22	34 (7)	34 [28, 41] (23, 47)	
	Missing	6				0				2			
**Practice status**	2C	5	22 (5)	24 [17-26] (15, 27)	0.804	13	236 (432)	42 [21, 273]	0.241	4	31 (7)	30[25, 38] (25, 39)	0.356
	Not 2C	54	23 (51)	22 [18-26] (4-52)		50	89 (133)	34 [11, 123]		59	35 (8)	34 [30, 40] (22, 74)	
	Missing	3				0				2			
**Number of children in practice aged ≤14 years (Correlation coefficient, Spearman’s Rho)**		59	-0.010,		0.941	63	-0.695		<0.001	63	-0.175		0.171
**Numbers of partners in the practice(Correlation coefficient, Spearman’s Rho)**		59	0.007,		0.958	63	-0.034		0.791	63	-0.070		0.588

^a^Data collected over 7 years were averaged. *N* = number of practices. T - test, analysis of variance and Spearman’s test (results expressed as Rho, equivalent to the correlation coefficient) were used for statistical analysis of comparisons in Grampian and Tayside. Mann Whitney, Kruskal Wallis and Spearman’s test were used for statistical analysis of comparisons in Highland due to the skewed distribution of referrals in Highland

### NHS Grampian

Univariate analyses identified the following variables as of interest: GP with a child health interest and deprivation, Table 2. In the multivariate model distance from hospital and deprivation were independently associated with practice referral rate, R^2^ = 0.38. With reference to practices closest to the hospital, referrals from practices which were intermediate and furthest from the hospital were reduced by -24/1000 [95 % CI -57, = 10] and − 51/1000 [-89, -13] respectively, *p* = 0.033 for trend. With reference to the most deprived two quintiles (SIMD 1 and 2 merged due to small numbers), referrals between 2011 and 2017 from practices in SIMD 3, 4 and 5 were reduced by -10/1000 [-63, + 42], -48/1000 [-99, + 3] and − 61 [-11, -112] *p* = 0.025 for trend.

### NHS Highland

**Fig. 1:**
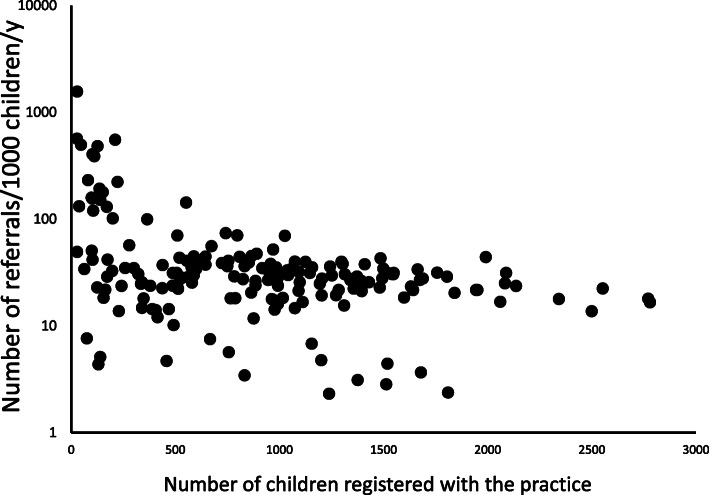
Scatter plot comparing the annual number of referrals of children to hospital outpatients/1000 children registered with the practice and the number of children registered with the practice. The relationship was significant (*p* < 0.001 in univariate and multivariate analyses)

The number of referrals/1000 was log transformed to achieve a near-normal distribution. In univariate analysis only the number of children was (inversely) related to the referral rate, *p* < 0.001 in a model where the R^2^ was 43 % (Fig. 1). To put the relationship between number of children registered with the practice and referrals into context, practices were placed into quintiles by number of children: the lowest quintile (median 16 children) had a median (IQR) of 1101 referrals/1000/y (35, 2980); the intermediate quintile (median 358 children) had a median (IQR) of 198 referrals/1000/y (100, 576); and the highest quintile (median 1393 children) had a median of 40 referrals/1000/y (20, 150).

### NHS Tayside

A GP with child health interest and number of children registered with the practice were considered but univariate analyses there was no significant relationship between number of referrals/1000 and these characteristics, Table 2.

### Multivariate model using data from all three Boards

In the merged database there were positive univariate correlations (Spearmans’ Rho) between number of partners and the number of children registered with the practice (0.504) and the number of GPs with special interest (0.146) and negative relationships with distance to hospital (-0.195) and deprivation (i.e. fewer partners in practices within more deprived communities, -0.215). Additionally, there was a negative relationship between the number of children registered with a practice and deprivation (-0.147). In the multivariate model, Table 3, the relationship between referral rate and number of children in the practice (inverse relationship *p* < 0.001) and number of partners in the practice (positive relationship *p* = 0.005) was significant. There was a trend which achieved significance for the referral rate to be higher from practices in more deprived compared to less deprived communities (Table 3).

**Table 3:** Output from a multivariate model whose outcome was number of referrals/1000children/y

		Relative % difference in referral rate/1000/year	*p* value
**Distance from hospital in tertiles**	1	1.01 [0.74, 1.37]	0.501
	2	1.25 [0.84, 1.85]	
	3	Reference	
**Deprivation Index (1 = most)**	1 and 2	1.55 [1.09, 2.21]	0.048
	3	1.26 [0.90, 1.77]	
	4 and 5	Reference	
**Practice status**	2 C	1.19 [0.79, 1.82]	0.399
	Not 2 C	Reference	
**Per 1000 children in practice aged ≤ 14 years**		-0.8 [-0.5, -1.1]	< 0.001
**Numbers of partners in the practice**		0.91 [0.86, 0.97]	0.005

Data from NHS Grampian, Highland and Tayside. The outcome was skewed (mean 60, standard deviation 142) and log transformed prior to analysis. The results are presented as relative difference. The model included a variable for the NHS board (*p* = 0.130). The model did not include GP with extended role in paediatrics since data were missing in 39 practices. The R^2^ for the model was 0.24

## Discussion

### Summary

This study was designed to describe associations between characteristics of individual primary care practices and the number of referrals to paediatric clinics across three Scottish health boards. Our premise was that characteristics of practices independent of the patient and their presenting complaint may be related to decision-making leading to referral. The main finding was when data from regions were combined, the number of children < 15 years of age, the number of partners and deprivation were associated with referral numbers. A secondary finding was that for individual boards, referral numbers were associated with different practice factors, and in two of the three health boards, one or two characteristics accounted for 38–51 % of variation between practices for the number of referrals made. Interventions aimed at delivering community-based integrated child health care need to be aware that different practices in different regions may have different needs.

### Strengths and Limitations

One strength of our study is that the findings add to what little is understood about what practice characteristics are associated with referral to paediatric clinics. A second strength is that we have analysed data from a large area where 15 % of children in Scotland live. Our study has several limitations which need to be acknowledged. First, we assumed that outcomes obtained at one point in time (e.g. presence of GP with a child health interest determined in 2018 and SIMD in 2016) were unchanged throughout the period where referral numbers were obtained (i.e. 2011–2017) but this may not be valid in all cases. Second, we assumed that the postcode of the practice would give a valid index of deprivation for the whole practice population and whilst this may be appropriate for practices in densely populated urban areas, this may not be as valid in more rural areas. Third, GP with child health interest status was missing in many practices and we have therefore not fully evaluated the association between the availability of GPs with child health interest and referral numbers from a practice. Finally, many factors which may influence referral numbers from a practice were not captured in our study, e.g. age and gender of partners, presence of advanced nurse practitioner, integration of care across primary and secondary care, and future research could consider these.

### Comparison with existing literature

Although there are no directly comparable studies of referrals to paediatric services, our results are consistent with some results of studies which analysed referrals to other specialties. For example, in NHS Grampian we observed that referrals were more likely from practices closer to the hospital, and this was also seen in a study from Wales [14]. In univariate (but not multivariate) analysis in NHS Grampian we observed an association between the presence of a GP with child health interest and an increased number referred. This association has been described previously [15], and this apparently unexpected result may reflect the different case mix seen by a GP with a child health interest, and also increased awareness of guidelines and “red flag” symptoms which prompt referral [6].

When data from all boards were analysed, there was a trend which achieved significance where practices in deprived communities had a higher *per capita* number of referrals than practices in less deprived communities and this is consistent with a study from 1997 [16], and a more recent qualitative study gives insight into the complexity of decision making leading to referrals from areas of high deprivation [17]. However when individual boards were considered, the relationship between referral rate and deprivation was heterogenous; in NHS Grampian referral numbers were relatively higher from more deprived communities, in NHS Tayside the reverse (non-significant) relationship was present and in NHS Highland there was no apparent socioeconomic gradient.

Health inequalities across the UK are well recognised. Understanding about health, health seeking behaviour and how to access to healthcare services differs across deprivation categories, and a recent report highlights how these inequalities persist [18].

The relationship between the number of children registered with a practice and referral rate was also heterogeneous across the three boards; overall there was an inverse relationship trend (Fig. 1) and this was most clearly seen for practices in NHS Highland but was not present at all in NHS Grampian practices. In contrast with our findings, a study of referrals to an eating disorder service found a positive relationship between number of patients registered with a practice and the number of referrals [19]. The relationship between number of registered patients and referrals seems complex and may be confounded by other factors, but in our analysis was independent of likely practice-level characteristics including deprivation and number of GPs working in the practice. Factors not measured in our study, such as the children’s age and presenting symptoms and gender of the referring clinician [20] may explain the negative relationship between the number of children registered with the practice and referral rate.

Unexpectedly we observed a positive between increased number of GPs working in a practice and the number of referrals, independent of the number of patients registered with the practice. There was no evidence of this association in the univariate analyses within individual boards, but the relationship between number of patients and number of GPs in a practice may have confounded the univariate analysis. It is possible that greater numbers of GPs could be associated with reduced personal continuity in primary care, a factor that is known to be associated with reduced paediatric referral rates [21]. The association between number of GPs and referral rates should nevertheless be interpreted with caution until verified in other populations.

There was also heterogeneity across the three boards for the relationship between having a GP with child health interest and referral rate; only in one board was there a significant relationship, where referral rate was higher from practices who had a GP with child health interest. A study from Norway reported that GPs with training in family medicine were less likely to refer patients to hospital for scheduled care [20]. As previously stated, a GP with child health interest may have greater awareness of red flags and referral pathways and this may lead to a higher referral rate. Our analysis was limited since information on GP with a child health interest was missing in 44 practices (23 %) so the association described should be interpreted with caution until replicated elsewhere.

We have shown that different practice-level factors shape the referrals within the Health Boards and this may be important in targeting interventions to address referral rates within and across regions.

There is no ideal number of referrals/1000 children, and although the child’s presenting symptom and/or sign is the major determinant of the decision to refer the child there are inevitably other determinants. Our observation that across all regions there was no consistent link between practice-level factor and number of referrals supports that paradigm that individual practice-level characteristics may not substantially affect referral numbers. However, within regions there was some variation in number of referrals between practices and in NHS Grampian and Highland between one third and half of this variation was explained by one or two practice-level characteristics. Whilst the associations we describe cannot prove causation, our results suggest that in some regions integrated work focussed on a small number of practice-level characteristics could reduce the number of scheduled referrals of children to outpatients. Further research could focus on exploring qualitative perspectives of clinicians, patients and carers of scheduled referral to hospital child health services. Wider data collection from the remaining health boards in Scotland and the NHS Trusts in the rest of the UK could be useful in establishing which characteristicss within regions are associated with differences in referral numbers between practices. This study has shown that factors other than illness influence decision making in relation to scheduled referrals to hospital-based child health services, they differ across regions and could be standardised to improve patient care. Finally, research could be focussed on developing and evaluating the impact of integrated community-based primary care paediatric scheduled care initiatives [22–25].

## Conclusions

Our study found that the number of referrals of children to medical outpatient departments was related to the number of patients registered with a practice (negative relationship), the number of GPs working in the practice (positive relationship) and deprivation in the community where the practice was located (positive relationship).

## Data Availability

The datasets generated during the current study are available from the corresponding author on reasonable request.

## Abbreviations

RACH: Royal Aberdeen Children’s Hospital
DGH: Dr Gray’s Hospital
SIMD: Scottish Index of Multiple Deprivation
